# Is loneliness associated with cancellation of medical appointments during the COVID-19 pandemic? Evidence from the Hamburg City Health Study (HCHS)

**DOI:** 10.1186/s12913-023-10490-y

**Published:** 2024-01-04

**Authors:** A. Hajek, E. Petersen, I. Schäfer, V. Harth, U. Koch-Gromus, M. Härter, H. Schulz, M. Scherer, H.H. König

**Affiliations:** 1 Department of Health Economics and Health Services Research, University Medical Center Hamburg-Eppendorf, Hamburg Center for Health Economics, Hamburg, Germany; 2 Department of Cardiology, University Heart and Vascular Center, University Medical Center Hamburg-Eppendorf, Hamburg, Germany; 3 Population Health Research Department, University Heart and Vascular Center, University Medical Center Hamburg-Eppendorf, Hamburg, Germany; 4 Institute for Occupational and Maritime Medicine (ZfAM), University Medical Center Hamburg-Eppendorf, Hamburg, Germany; 5 Department of Medical Psychology, University Medical Center Hamburg-Eppendorf, Hamburg, Germany; 6 Department of General Practice and Primary Care, University Medical Center Hamburg-Eppendorf, Hamburg, Germany

**Keywords:** COVID-19, Corona-virus, Postponed healthcare use, Healthcare use

## Abstract

**Background:**

The COVID-19 pandemic engendered numerous societal and economic challenges in addition to health-related concerns. Maintenance of healthcare utilization assumed immense significance during this period. However, few studies have examined the association between loneliness and cancelled medical appointments during the COVID-19 pandemic. This study aimed to examine whether medical appointments are less likely to be cancelled with increased loneliness during a pandemic. We analyzed the association between loneliness and both patient- and provider-initiated appointment cancellations.

**Methods:**

Cross-sectional data from the Hamburg City Health Study (HCHS) were collected during April 2020–November 2021. The analytical sample included 1,840 participants with an average age of 55.1 years (standard deviation: 6.5, range 45–76 years). Medical appointments cancelled by individuals—medical appointments in general, and GP, specialist, and dentist appointments—and appointments cancelled by healthcare providers served as outcome measures. Loneliness was quantified using a single item ranging from 0 to 10. Accordingly, we created empirical loneliness tertiles. Covariates were selected based on the Andersen model. Several penalized maximum likelihood logistic regressions were utilized to examine the association between loneliness and cancellation of medical appointments during the COVID-19 pandemic.

**Results:**

The penalized maximum likelihood logistic regressions showed that, compared to individuals in the lowest loneliness tertiles, individuals in the other two tertiles reported a higher chance of medical appointments cancellation by individuals, particularly driven by cancelled GP appointments. Except for age and sex, none of the covariates were comparably associated with the outcomes. When appointments cancelled by healthcare providers served as outcomes, only a higher number of chronic conditions was significantly positively associated with it.

**Conclusions:**

Individuals scoring higher in loneliness had a greater chance of cancelling medical (particularly GP) appointments. This may contribute to a potential cascade of loneliness and skipped medical appointments in the future, resulting in adverse health outcomes over the medium-to-long term. Future research should examine whether lonely people are more likely to lack the social motivation to visit the doctor.

**Supplementary Information:**

The online version contains supplementary material available at 10.1186/s12913-023-10490-y.

## Background

The COVID-19 pandemic engendered numerous societal and economic challenges in addition to significant health-related concerns. Maintenance of healthcare utilization assumed immense significance during this period. The utilization of medical appointments is key to staying healthy. However, studies have shown that during the pandemic, individuals postponed the utilization of preventive services, such as cancer screenings and health checkups, and avoided dental visits, even in cases of pain [1–3].

Additionally, the enforcement of temporary physical distancing induced loneliness among people (i.e., perceived discrepancy between current social relationships and desired social relationships [4]). Loneliness is a crucial social challenge and can contribute to morbidity and mortality [5, 6]. High prevalence rates of loneliness were identified in Germany during the pandemic [7]. Studies have emphasized it as a serious challenge—nationally as well as globally [8].

After adjusting for health-related factors, we expected an association between a higher level of loneliness and a lower chance of individuals cancelling medical appointments. This could be explained by the notion that lonely individuals may seek to compensate for their potential lack of social contact, especially during the pandemic, by visiting physicians [9]. Alternatively, the actual use of physicians could at least complement a few private contacts. People with high levels of loneliness could perceive the office of a general practitioner (GP) as a place of (social) exchange.

However, there exist reasons to suspect an association between higher loneliness and a higher chance of cancelling medical appointments. Particularly, one could assume that an increase in loneliness may induce further social withdrawal [10]. Contact with and visits to GPs could become increasingly limited over time. However, overall, we assume that the idea of substituting or supplementing a lack of social contact with GP visits—as discussed above—outweighs the potential phenomenon of greater social withdrawal.

To date, there has been a lack of research on the association between loneliness and cancelled medical appointments during the COVID-19 pandemic. A somewhat-related recent study showed that not living alone was associated with a higher possibility of cancelled/postponed healthcare services among patients with non-communicable diseases in southern Italy [11]. However, it is noteworthy that living arrangement is only partly related to feeling lonely. Therefore, our aim was to address this knowledge gap using data from Germany. Such knowledge is crucial for identifying individuals at risk of cancelled medical appointments, which could have a negative impact on their health in the mid- and long-term. In summary, these findings could help prevent the issue of unmet health needs, and thus, possibly contribute to long-term health maintenance.

To understand the context of this study, it is essential to outline the key characteristics of the German healthcare system. Health insurance is mandatory in Germany, with the majority of individuals—approximately nine out of 10—being members of the social statutory health insurance (SHI); only one out of 10 individuals has private health insurance (PHI). Civil servants, employed individuals with higher income, or self-employed individuals typically opt for a PHI. Both types of health insurances cover most outpatient treatment expenses, including visits to GPs, specialists, and dentists, thus ensuring access to healthcare for all insured individuals. Waiting period for access to healthcare is relatively short in Germany [12, 13]; further details of the German healthcare system can be found in Passon et al. [14].

## Methods

### Sample

The Hamburg City Health Study (HCHS) is a population-based observational study conducted at the University Medical Center Hamburg-Eppendorf (UKE) since 2016 (in detail: [15]). Hamburg is the second most populous city in Germany, with more than 1.8 million inhabitants.

It comprises seven districts and 104 urban quarters, accommodating people from diverse social backgrounds. Although Hamburg is predominantly urban, it also includes rural areas. Of the city’s residents, more than 0.8 million are aged 45 years or older. Hamburg’s demographic composition differs from that of Germany in several ways. Specifically, Hamburg is characterized by a higher proportion of people of different nationalities, a higher proportion of single people, and a higher proportion of residents with higher educational qualifications and income (see [15]).

More than 30 UKE clinics and institutes collaborate to collect and analyze various risk factors for common diseases, such as heart attack, depression, cancer, stroke, and dementia. The key aims of the HCHS were to: (1) ascertain the factors contributing to the emergence of functional health limitations and major chronic conditions, (2) examine the predictive factors influencing the survival of individuals with chronic conditions, and (3) identify the factors that bolster life in individuals who have survived major chronic illnesses [15].

A random sample stratified by age and sex was drawn from the residents’ registration office to include participants from the general population of Hamburg, aged 45 to 74 years at the time of sampling. Individuals with insufficient language or cognitive abilities to complete the questionnaires, as well as those with physical limitations that prevented them from participating in the seven-hour examination program at the study center, were excluded. As of August 2022, more than 17,000 participants underwent examinations.

In the HCHS, participants underwent a comprehensive assessment comprising 13 established and five innovative examinations focused on the function and structure of major organ systems. Additionally, the evaluation incorporated self-reported information through questionnaires on various aspects of the participants’ lives, such as, lifestyle, environment, diet, physical activity, sexual health, work life, psychosocial factors, quality of life, digital media usage, medical and family history, and healthcare utilization [15].

In April 2020, during the pandemic, a collaboration was established between the Free and Hanseatic City of Hamburg and an interdisciplinary consortium of the UKE. As a result of this funding project, the HCHS integrated the COVID-19 module, which includes, among other things, targeted questions specifically related to COVID-19 (such as the outcomes presented in the next section).

This was a cross-sectional, observational study. All consecutive HCHS participants between April 2020 and November 2021 received the COVID-19 questionnaire. The inclusion and exclusion criteria corresponded to those of the HCHS (see also: [16]). In sum, n = 1,840 persons were included in the analytical sample. The translated (i.e., English version) dependent variables and translated independent variable of interest (loneliness) are shown in Supplementary File 1.

All participants gave their written informed consent to participate in the study and to the analysis of the collected data. This study was approved by the Ethics Committee of the Hamburg Medical Association (PV5113).

### Dependent variables

The questions regarding the utilization of healthcare services during the COVID-19 pandemic are similar to those used in other large-scale studies [1, 17, 18]. Participants were first asked (1) whether they had personally chosen not to visit a physician despite scheduled appointments or experiencing symptoms. If they responded affirmatively, additional information was gathered about the specific types of appointments that were postponed across various medical fields, such as, (2) general practitioners, (3) specialists, and (4) dentists. Moreover, the participants were asked if they had refrained from (5) seeking treatment in the emergency room, even in the case of a medical emergency. Owing to the small number of cases, only variables (1) to (4) served as separate outcome measures (see the statistical analysis section).

Moreover, the participants were queried about (1) any cancellation or rescheduling of planned appointments by healthcare providers. Thereafter, they were prompted to indicate the nature of these appointments, including whether these were (2) specialist consultations, (3) general practitioner visits, (4) dental appointments, (5) hospital treatments or surgeries, (6) inpatient rehabilitation programs, (7) psychotherapeutic sessions, or (8) other therapeutic treatments. However, owing to the small number of cases, only the general cancellation of planned appointments by healthcare providers (1) served as an additional outcome measure. For each outcome, individuals should refer to the period from February 2020 onward.

### Independent variable of interest: loneliness

Loneliness was quantified using a single-item measure. Individuals reported how lonely they feel at the moment on a scale ranging from 0 (not at all) to 10 (extremely). Only the endpoints were labelled. Based on this, empirical loneliness tertiles were formed. Single items are commonly used to assess loneliness [19]. Furthermore, previous research has indicated the sensitivity of this measurement and its strong correlation with the UCLA loneliness scale [20]. The reliability and validity of this measure have also been demonstrated in other research [19].

### Covariates

Covariates were selected based on the (extended [21]) model of healthcare use developed by Andersen [22]. This model divides predisposing characteristics (e.g., sex or age), enabling resources (e.g., health insurance status or income), and need factors (e.g., chronic conditions). Notably, in accordance with the extended model, loneliness can be treated as a psychosocial factor (in addition to the predisposing characteristics, enabling resources, and need factors).

Regarding the predisposing characteristics, we included the following in the regression analysis: age in years, sex (male and female), and marital status (married, living together with spouse; married, living separated from spouse; single; divorced; and widowed). Marital status was dichotomized (1 = married, living together with spouse; 0 = otherwise).

Regarding the enabling resources, we included in the regression analysis: household net income (17 categories) and health insurance status (statutory health insurance; other [e.g., including private health insurance]). The 17 income categories include: 1 = Under 500 €, 2 = 500 € to under 750 €, 3 = 750 € to under 1,000 €, 4 = 1,000 € to under 1,250 €, 5 = 1,250 € to under 1,500 €, 6 = 1,500 € to under 1,750 €, 7 = 1,750 € to under 2,000 €, 8 = 2,000 € to under 2,250 €, 9 = 2,250 € to under 2,500 €, 10 = 2,500 € to under 3,000 €, 11 = 3,000 € to under 3,500 €, 12 = 3,500 € to under 4,000 €, 13 = 4,000 € to under 4,500 €, 14 = 4,500 € to under 5,000 €, 15 = 5,000 € to under 6,000 €, 16 = 6,000 € to under 8,000 €, 17 = 8,000 € or higher. Based on this, we formed empirical income tertiles.

Regarding the need factors, we included a score for chronic conditions in the regression analysis. To this end, a count score for the chronic conditions (for each chronic condition: 0 = absence of the condition; 1 = presence of the condition) was calculated. The 14 chronic conditions included: arterial hypertension, diabetes mellitus, atrial fibrillation, stroke, heart failure, dementia, bronchial asthma, chronic bronchitis/COPD, myocardial infarction, atrial fibrillation, coronary artery disease, angina pectoris, cancer, and kidney disease.

### Statistical analysis

Sample characteristics were provided for the total analytical sample and stratified by medical appointments cancelled by individuals (no or yes) and appointments cancelled by healthcare providers (no or yes). Subsequently, we used several penalized maximum likelihood logistic regressions [23, 24] (using the Stata tool: “firthlogit” [25]). To mitigate the small-sample bias caused by the limited case numbers for certain variables, the Firth method [23] was employed. More precisely, this approach was selected to address the potential statistical challenges arising from the small sample size. List-wise deletion was applied to address the missing values. In the sensitivity analysis, the number of individuals in the household (including the respondent) was added to the main regression model.

Outcome measures included: cancelled medical appointments in general, cancelled GP appointments, cancelled specialist appointments, and cancelled dentist appointments. Appointments cancelled by *healthcare providers* served as the outcome measure.

The level of statistical significance was set at *p* < 0.05. P-values within the range of 0.05–0.10 were regarded as marginally significant. All statistical analyses were conducted utilizing the Stata 16.1 (Stata Corp., College Station, Texas, USA).

## Results

### Sample characteristics

Table 1 presents the sample characteristics for our analytical sample (with medical appointment in general as outcome) (n = 1,840), stratified by medical appointments cancelled by individuals as well as those by healthcare providers. The average age was 55.1 years (SD: 6.5 years; 45–76 years), with approximately 45% of the individuals being female and 60% of the individuals being married and living together with their spouse. The average number of chronic conditions equaled 1.2 (SD: 1.2). In sum, 15.3% of the individuals cancelled medical appointments (most frequently, both specialist and dentist appointments, followed by cancelled GP appointments). Moreover, 7.2% of the individuals reported appointments cancelled by healthcare providers. Further details are presented in Table 1.

**Table 1:** Sample characteristics for the analytical sample (with medical appointments in general as outcome; n = 1,840 individuals)

	Medical appointments in general (cancelled by individuals)			Appointments cancelled by healthcare providers	
	No, not cancelled	Yes, cancelled	Total	No, not cancelled	Yes, cancelled
	N = 1558	N = 282	N = 1840	N = 1702	N = 132
GP appointment: N (%)					
No, not cancelled	1558 (100.0%)	228 (80.9%)	1786 (97.1%)	1659 (97.5%)	121 (91.7%)
Yes, cancelled	0 (0.0%)	54 (19.1%)	54 (2.9%)	43 (2.5%)	11 (8.3%)
Specialist appointment: N (%)					
No, not cancelled	1558 (100.0%)	165 (58.5%)	1723 (93.6%)	1607 (94.4%)	110 (83.3%)
Yes, cancelled	0 (0.0%)	117 (41.5%)	117 (6.4%)	95 (5.6%)	22 (16.7%)
Dentist appointment: N (%)					
No, not cancelled	1558 (100.0%)	167 (59.2%)	1725 (93.8%)	1605 (94.3%)	114 (86.4%)
Yes, cancelled	0 (0.0%)	115 (40.8%)	115 (6.3%)	97 (5.7%)	18 (13.6%)
Loneliness: N (%)					
Lowest tertile	764 (49.0%)	105 (37.2%)	869 (47.2%)	813 (47.8%)	55 (41.7%)
Second tertile	362 (23.2%)	82 (29.1%)	444 (24.1%)	408 (24.0%)	35 (26.5%)
Highest tertile	432 (27.7%)	95 (33.7%)	527 (28.6%)	481 (28.3%)	42 (31.8%)
Sex: N (%)					
Male	885 (56.8%)	128 (45.4%)	1013 (55.1%)	947 (55.6%)	64 (48.5%)
Female	673 (43.2%)	154 (54.6%)	827 (44.9%)	755 (44.4%)	68 (51.5%)
Age: Mean (SD)	55.3 (6.6)	54.1 (6.1)	55.1 (6.5)	55.1 (6.5)	55.0 (6.8)
Marital status: N (%)					
Married, living separated from spouse; single; divorced; widowed	611 (39.2%)	118 (41.8%)	729 (39.6%)	673 (39.5%)	54 (40.9%)
Married, living together with spouse	947 (60.8%)	164 (58.2%)	1111 (60.4%)	1029 (60.5%)	78 (59.1%)
Household net income: N (%)					
Lowest tertile	614 (39.4%)	117 (41.5%)	731 (39.7%)	667 (39.2%)	60 (45.5%)
Second tertile	586 (37.6%)	104 (36.9%)	690 (37.5%)	643 (37.8%)	45 (34.1%)
Highest tertile	358 (23.0%)	61 (21.6%)	419 (22.8%)	392 (23.0%)	27 (20.5%)
Health insurance: N (%)					
Statutory health insurance	1249 (80.2%)	230 (81.6%)	1479 (80.4%)	1361 (80.0%)	112 (84.8%)
Other	309 (19.8%)	52 (18.4%)	361 (19.6%)	341 (20.0%)	20 (15.2%)
Number of chronic conditions: Mean (SD)	1.2 (1.2)	1.2 (1.1)	1.2 (1.2)	1.2 (1.1)	1.5 (1.4)

### Regression analysis

The penalized maximum likelihood logistic regression with medical appointments cancelled by *individuals* is shown in Table 2. The outcome measures included cancelled medical appointments in general, cancelled GP appointments, cancelled specialist appointments, and cancelled dentist appointments (in each case: 0 = no, not cancelled; 1 = yes, cancelled).

**Table 2:** Determinants of medical appointments cancelled by patients (0 = no, not cancelled; 1 = yes, cancelled) since February 2020. Findings of penalized maximum likelihood logistic regressions

	(1)	(2)	(3)	(4)
Independent variables	Medical appointment in general	GP appointment	Specialist appointment	Dentist appointment
Loneliness: - Second tertile (Reference category: Lowest tertile)	1.59**	2.23*	1.48+	1.74*
	(1.15–2.18)	(1.11–4.47)	(0.94–2.35)	(1.10–2.74)
- Highest tertile	1.54**	2.41*	1.30	1.39
	(1.13–2.10)	(1.23–4.73)	(0.82–2.07)	(0.87–2.22)
Sex: Female (Reference category: Male)	1.55**	1.23	1.80**	1.42+
	(1.19–2.01)	(0.71–2.14)	(1.22–2.65)	(0.96–2.08)
Age in years	0.97**	0.98	0.97*	0.99
	(0.95–0.99)	(0.94–1.03)	(0.94–1.00)	(0.96–1.02)
Marital status: Married, living together with spouse (Reference category: Other)	1.06	0.99	0.91	0.90
	(0.79–1.42)	(0.54–1.83)	(0.60–1.40)	(0.59–1.39)
Household net income: - Second tertile (Reference category: Lowest tertile)	0.98	1.21	1.25	1.21
	(0.71–1.34)	(0.63–2.34)	(0.79–1.99)	(0.74–1.97)
- Highest tertile	0.92	0.75	1.00	1.68+
	(0.62–1.38)	(0.30–1.87)	(0.54–1.83)	(0.94–2.98)
Health insurance: Other (Reference category: Statutory health insurance)	1.06	1.50	0.99	1.12
	(0.74–1.51)	(0.74–3.04)	(0.58–1.69)	(0.69–1.84)
Number of chronic conditions	1.06	1.15	1.08	1.03
	(0.94–1.19)	(0.93–1.43)	(0.91–1.27)	(0.86–1.22)
Constant	0.59	0.03**	0.23	0.08**
	(0.17–2.00)	(0.00–0.39)	(0.04–1.41)	(0.01–0.47)
Observations	1,840	1,840	1,840	1,840

Results include Odds Ratios, presented with corresponding 95% confidence intervals (CI); *** *p* < 0.001, ** *p* < 0.01, * *p* < 0.05, + *p* < 0.10

Regressions showed that being in the second loneliness tertile (compared to the lowest loneliness tertile) was significantly associated with a higher chance of cancelled medical appointment in general (OR: 1.59, 95% CI: 1.15–2.18), cancelled GP appointments (OR: 2.23, 95% CI: 1.11–4.47), and cancelled dentist appointments (OR: 1.74, 95% CI: 1.10–2.74). Moreover, a marginal significant association with cancelled specialist appointments have been identified (OR: 1.48, 95% CI: 0.94–2.35). Additionally, being in the highest loneliness tertile (compared to the lowest loneliness tertile) was significantly associated with a higher chance of cancelled medical appointment in general (OR: 1.54, 95% CI: 1.13–2.10) and cancelled GP appointments (OR: 2.41, 95% CI: 1.23–4.73). Notably, only the two predisposing characteristics of younger age and being female (compared to being male) were associated with a higher chance of cancelled medical appointments in general and cancelled specialist appointments, whereas neither the enabling resources nor the need factor were significantly associated with the outcomes.

We examined the determinants of appointments cancelled by *healthcare providers* using the penalized maximum likelihood logistic regression (see Table 3). In this case, only the cancelled appointments in general served as outcome measures because of the small number of cases (e.g., for GPs; see the Methods section). In this case, the loneliness tertiles were not significantly associated with appointments cancelled by healthcare providers. Furthermore, neither the predisposing characteristics nor the enabling resources were significantly associated with the outcome. Only a higher number of chronic conditions was significantly associated with a higher chance of appointments being cancelled by healthcare providers.

**Table 3:** Determinants of appointments cancelled by healthcare providers (0 = no, not cancelled; 1 = yes, cancelled) since February 2020. Findings of penalized maximum likelihood logistic regression

	(1)
Independent variables	Healthcare providers in general
Loneliness: - Second tertile (Reference category: Lowest tertile)	1.25
	(0.80–1.94)
- Highest tertile	1.22
	(0.80–1.88)
Sex: Female (Reference category: Male)	1.29
	(0.90–1.85)
Age in years	0.98
	(0.96–1.01)
Marital status: Married, living together with spouse (Reference category: Other)	1.09
	(0.73–1.64)
Household net income: - Second tertile (Reference category: Lowest tertile)	0.82
	(0.53–1.27)
- Highest tertile	0.89
	(0.51–1.56)
Health insurance: Other (Reference category: Statutory health insurance)	0.82
	(0.49–1.39)
Number of chronic conditions	1.22**
	(1.06–1.40)
Constant	0.59
	(0.17–2.00)
Observations	1,835

Results include Odds Ratios, presented with corresponding 95% confidence intervals (CI); *** *p* < 0.001, ** *p* < 0.01, * *p* < 0.05, + *p* < 0.10

In the first sensitivity analysis, we added age-squared to our regression model. However, the association between loneliness and the different outcomes remained virtually identical. In the next sensitivity analysis, the number of household members was added to the regression model, and the significant association between loneliness and cancelled GP appointments disappeared. For further details, please see the Supplementary Files 2 and 3.

## Discussion

We examined the association between loneliness and cancelled medical appointments during the pandemic based on the HCHS data. Our study showed that individuals with higher levels of loneliness had a higher chance of cancelling medical (particularly GP) appointments.

It is worth reiterating that it is difficult to compare our findings with those of previous studies as this is the first study focusing on the association between loneliness and *cancelled* medical appointments *during the pandemic*. However, a previous meta-analysis showed that individuals experiencing loneliness had more *actual* GP visits [26] prior to the pandemic. Conversely, a study conducted during the pandemic (data collected in March 2022) showed that lower loneliness levels were associated with a higher number of GP visits among the general adult population in Germany [27]. Our results are similar to those of the latter study [27].

Our findings contradict our initial expectations, that lonely people attend medical appointments to remain in touch and compensate for the lack of personal contact during the pandemic. Our findings reflect the notion that loneliness may lead to social withdrawal during a pandemic. We assume that this withdrawal increases the chance of cancelling medical appointments. Moreover, this may be attributed to the fact that individuals experiencing greater loneliness may experience a lack of motivation during a pandemic [27]. Loneliness could manifest as poor mental health and lack of motivation, which may lead to the tendency to cancel GP appointments.

Interestingly, loneliness was not significantly associated with appointments cancelled by healthcare providers, whereas chronic conditions was significantly positively associated with this outcome. From our perspective, this also illustrates that loneliness of individuals is mainly related to the decision to cancel on the part of the individuals and not on the part of healthcare providers. Such findings are plausible because, from the perspective of a healthcare provider, loneliness is, probably, only indirectly related to health. Moreover, cancellations by healthcare providers are frequently organizational in nature and the loneliness scores of patients are (commonly) unknown to healthcare providers. Conversely, chronic conditions such as cancer could be directly associated with a severe course of the disease (in case of COVID-19 infection). Therefore, healthcare providers may have cancelled appointments—and replaced them, as far as possible, with telemedicine services—to mitigate the risk of COVID-19 infection among those patients [28]. Another possible explanation is that non-acute appointments were postponed—perhaps in many cases, chronically ill patients were affected—whereas acute COVID-19 cases were prioritized. The same has been observed during hospital surgeries [29].

Regarding the other covariates, the predisposing characteristics of being female and younger were associated with a higher chance of cancelling medical appointments. This may reflect the findings that both groups—that is, younger individuals and female individuals—report higher COVID-19-induced anxiety among the general adult population in Germany [30]. Higher COVID-19-induced anxiety may contribute to an increased chance of cancelling medical appointments [1].

Regarding strengths and limitations, the data were obtained from the acknowledged HCHS. Detailed information on non-utilization was collected during the pandemic. Covariates were selected using the Andersen model. One limitation of this study is that the chronic conditions were assessed during the baseline assessment. Therefore, information on the *current* self-rated and objective health is lacking. Moreover, the number and types of services that were cancelled or postponed remain unknown. Additionally, this study used a cross-sectional design, which limited its ability to draw causal inferences. The participants answered these questions independently. Therefore, recall bias could not be ruled out. Moreover, a potential selection bias could not be excluded. Finally, Hamburg differs from the German population in a few respects (see the Methods section for details).

## Conclusion

This study revealed that individuals with higher levels of loneliness were more likely to cancel their medical appointments, especially those with GPs. This finding raises concerns as it could potentially lead to a chain reaction of increased loneliness and more postponed/cancelled medical appointments in the future. Consequently, there is a potential risk of adverse health outcomes over the medium-to-long term, which should to be addressed. To mitigate these potential negative health consequences, future research should investigate whether loneliness attenuates the social support that motivates individuals to seek medical care. Additionally, the extent to which loneliness will result in the cancellation of visits to doctors post-pandemic is a topic of future research.

### Electronic supplementary material

Below is the link to the electronic supplementary material.

Supplementary Material 1

Supplementary Material 2

Supplementary Material 3

## Data Availability

The datasets generated and/or analyzed during the current study are not publicly available because of legal restrictions but are available from the corresponding author upon reasonable request.

## Abbreviations

COPD: Chronic obstructive pulmonary disease
GP: General practitioner
HCHS: Hamburg City Health Study
OR: Odds ratio
PHI: Private health insurance
SD: Standard deviation
SHI: Statutory health insurance
UCLA: University of California, Los Angeles
UKE: University Medical Center Hamburg-Eppendorf
