# Supplementary material for: Is loneliness associated with cancellation of medical appointments during the COVID-19 pandemic? Evidence from the Hamburg City Health Study (HCHS)
Source: BMC Health Serv Res. 2024 Jan 4;24:32. doi: 10.1186/s12913-023-10490-y (PMC10768441; doi:10.1186/s12913-023-10490-y)
Supplement: Supplementary file 1 — Supplementary Material 1 [file 12913_2023_10490_MOESM1_ESM.docx]

Supplementary File 1. Translated dependent variables and the translated independent variable of interest (loneliness)

**Translated dependent variables**

Have you yourself gone without a doctor's visit since February 2020, even though it was planned or you had complaints?

☐ Yes ☐ No (if no, please skip the next question)

If yes, what did you cancel or not avail yourself of? (Multiple answers possible)

☐ I cancelled an upcoming check-up appointment with the GP

☐ I have cancelled an upcoming check-up appointment with a specialist doctor

☐ I have cancelled an upcoming check-up appointment with the dentist

☐ I have refrained from using an ambulance or emergency room despite a medical emergency in the following situation:

__________________________________________________________________

☐ Other situation. Please specify:

__________________________________________________________________

Has a doctor or hospital postponed a planned doctor's visit or other planned treatment since February 2020?

☐ Yes ☐ No (if no, please skip the next question)

If yes, what was cancelled by the doctor or hospital? (Multiple answers possible)

☐ An agreed check-up appointment with a specialist was cancelled by the practice

☐ An agreed check-up appointment with the general practitioner was cancelled by the practice

☐ An arranged check-up appointment with the dentist was cancelled by the practice

☐ A planned hospital treatment or operation was postponed

☐ A planned inpatient rehabilitation measure was postponed

☐ A psychotherapeutic treatment was suspended or postponed

☐ A physiotherapeutic, occupational therapy or speech therapy treatment was suspended or postponed

☐ Other cancellations or postponements. Which ones?

__________________________________________________________________

**Translated independent variable of interest (loneliness)**

How lonely do you feel at the moment?

(from 0 = not at all to 10 = extremely)
