# Supplementary material for: Is loneliness associated with cancellation of medical appointments during the COVID-19 pandemic? Evidence from the Hamburg City Health Study (HCHS)
Source: BMC Health Serv Res. 2024 Jan 4;24:32. doi: 10.1186/s12913-023-10490-y (PMC10768441; doi:10.1186/s12913-023-10490-y)
Supplement: Supplementary file 2 — Supplementary Material 2 [file 12913_2023_10490_MOESM2_ESM.docx]

Supplementary File 2. Determinants of medical appointments cancelled by patients (0 = no, not cancelled; 1 = yes, cancelled) since February 2020. Findings of penalized maximum likelihood logistic regressions, additionally adjusting for the number of household members

|  | (1) | (2) | (3) | (4) |
| --- | --- | --- | --- | --- |
| Independent variables | Medical appointment in general | GP appointment | Specialist appointment | Dentist appointment |
|  |  |  |  |  |
| Loneliness: - Second tertile (Reference category: Lowest tertile) | 1.45* | 1.52 | 1.70+ | 1.55 |
|  | (1.00 - 2.10) | (0.68 - 3.41) | (1.00 - 2.91) | (0.91 - 2.65) |
| - Highest tertile | 1.40+ | 1.71 | 1.40 | 1.35 |
|  | (0.97 - 2.01) | (0.80 - 3.69) | (0.81 - 2.42) | (0.79 - 2.33) |
| Sex: Female (Reference category: Male) | 1.42* | 1.10 | 1.61* | 1.38 |
|  | (1.05 - 1.93) | (0.57 - 2.12) | (1.03 - 2.52) | (0.88 - 2.17) |
| Age in years | 0.97* | 0.99 | 0.96+ | 0.98 |
|  | (0.94 - 0.99) | (0.94 - 1.05) | (0.92 - 1.00) | (0.94 - 1.02) |
| Marital status: Married, living together with spouse (Reference category: Other) | 0.97 | 0.73 | 0.83 | 1.15 |
|  | (0.66 - 1.41) | (0.33 - 1.63) | (0.47 - 1.45) | (0.65 - 2.04) |
| Household net income: - Second tertile (Reference category: Lowest tertile) | 1.03 | 0.75 | 1.22 | 1.41 |
|  | (0.70 - 1.50) | (0.34 - 1.67) | (0.70 - 2.12) | (0.78 - 2.56) |
| - Highest tertile | 1.01 | 0.59 | 1.03 | 2.02* |
|  | (0.63 - 1.64) | (0.20 - 1.72) | (0.50 - 2.14) | (1.01 - 4.06) |
| Health insurance: Other (Reference category: Statutory health insurance) | 1.12 | 1.17 | 0.91 | 1.20 |
|  | (0.75 - 1.67) | (0.48 - 2.85) | (0.49 - 1.69) | (0.69 - 2.09) |
| Number of chronic conditions | 1.05 | 1.17 | 1.08 | 1.05 |
|  | (0.92 - 1.19) | (0.91 - 1.50) | (0.89 - 1.30) | (0.86 - 1.27) |
| Number of household members | 0.96 | 1.34 | 0.98 | 0.86 |
|  | (0.81 - 1.14) | (0.94 - 1.90) | (0.76 - 1.26) | (0.66 - 1.12) |
| Constant | 0.86 | 0.02* | 0.40 | 0.14 |
|  | (0.17 - 4.36) | (0.00 - 0.59) | (0.03 - 4.60) | (0.01 - 1.63) |
|  |  |  |  |  |
| Observations | 1,360 | 1,360 | 1,360 | 1,360 |

Results include Odds Ratios, presented with corresponding 95% confidence intervals (CI); *** p<0.001, ** p<0.01, * p<0.05, + p<0.10
