# Supplementary material for: Is loneliness associated with cancellation of medical appointments during the COVID-19 pandemic? Evidence from the Hamburg City Health Study (HCHS)
Source: BMC Health Serv Res. 2024 Jan 4;24:32. doi: 10.1186/s12913-023-10490-y (PMC10768441; doi:10.1186/s12913-023-10490-y)
Supplement: Supplementary file 3 — Supplementary Material 3 [file 12913_2023_10490_MOESM3_ESM.docx]

Supplementary File 3. Determinants of medical appointments cancelled by patients (0 = no, not cancelled; 1 = yes, cancelled) since February 2020. Findings of penalized maximum likelihood logistic regression, additionally adjusting for the number of household members

|  | (1) |
| --- | --- |
| Independent variables | Healthcare providers in general |
|  |  |
| Loneliness: - Second tertile (Reference category: Lowest tertile) | 1.24 |
|  | (0.73 - 2.08) |
| - Highest tertile | 1.15 |
|  | (0.69 - 1.91) |
| Sex: Female (Reference category: Male) | 1.23 |
|  | (0.80 - 1.88) |
| Age in years | 0.98 |
|  | (0.95 - 1.02) |
| Marital status: Married, living together with spouse (Reference category: Other) | 1.00 |
|  | (0.59 - 1.71) |
| Household net income: - Second tertile (Reference category: Lowest tertile) | 0.74 |
|  | (0.44 - 1.27) |
| - Highest tertile | 0.99 |
|  | (0.51 - 1.90) |
| Health insurance: Other (Reference category: Statutory health insurance) | 0.88 |
|  | (0.49 - 1.60) |
| Number of chronic conditions | 1.26** |
|  | (1.08 - 1.48) |
| Number of household members | 1.00 |
|  | (0.78 - 1.28) |
| Constant | 0.13+ |
|  | (0.01 - 1.21) |
|  |  |
| Observations | 1,356 |

Results include Odds Ratios, presented with corresponding 95% confidence intervals (CI); *** p<0.001, ** p<0.01, * p<0.05, + p<0.10
